# Lectures on Venerial Diseases

**Published:** 1864-11

**Authors:** 


LECTURES ON VENERIAL DISEASES. By WILLIAM A. HAMMOND, M.D. Philadelphia: J. B. LIPPINCOTT & Co. 1864.

This volume is published in excellent style, and embraces 20 lectures on the nature, symptoms, and treatment of the various forms of syphilitic disease. We have not had time to examine its contents in detail; but have no doubt it will amply repay the practitioner for a perusal.

For sale by S. C. Griggs & Co., 41 Lake Street, Chicago.

